# Supplementary figures and images for: Comparative Genomic Analysis of Three Salmonid Species Identifies Functional Candidate Genes Involved in Resistance to the Intracellular Bacterium Piscirickettsia salmonis
Source: Front Genet. 2019 Aug 5;10:665. doi: 10.3389/fgene.2019.00665 (PMC6690157; doi:10.3389/fgene.2019.00665)

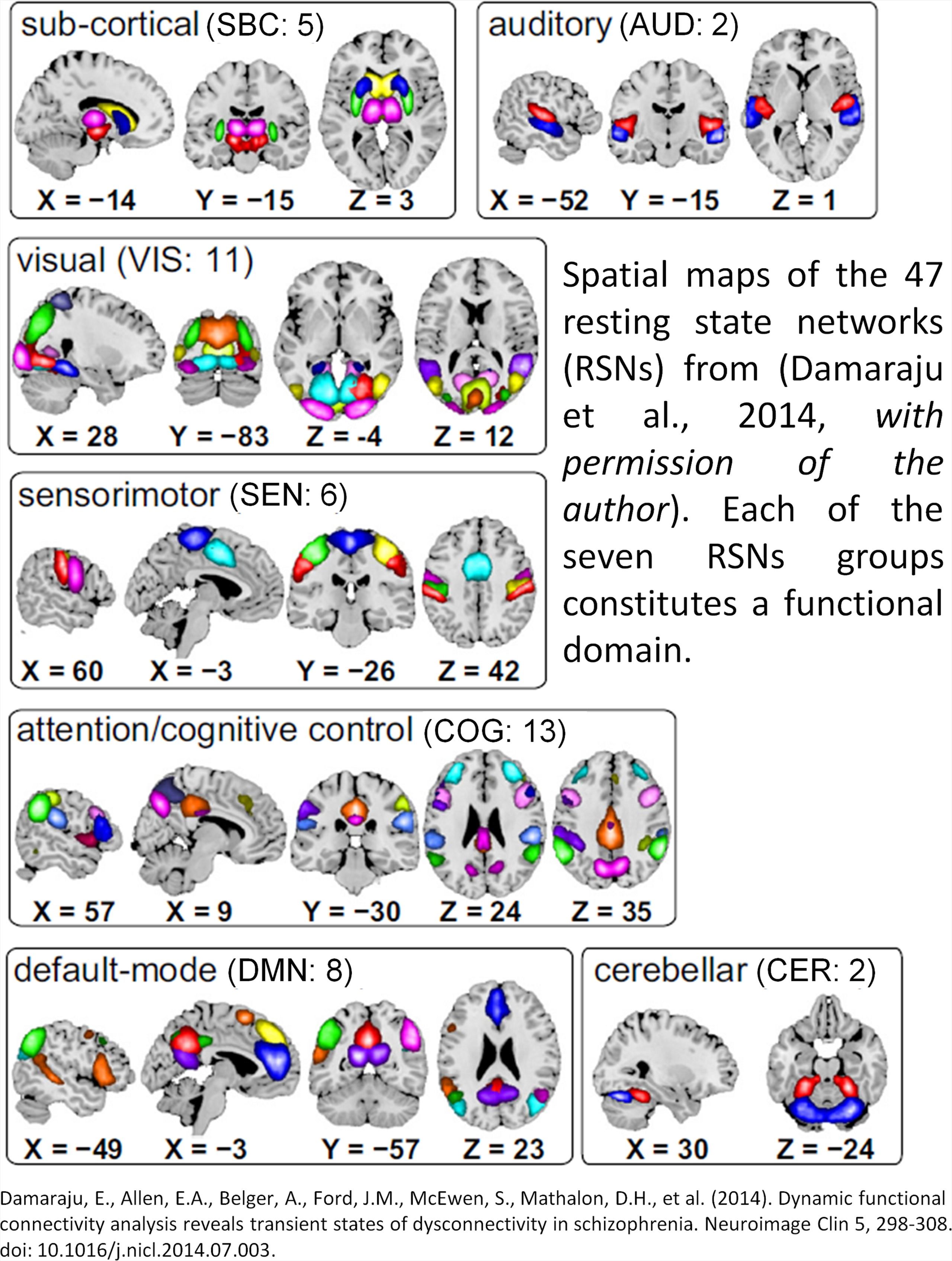

Supplement: Figure S1 — Manhattan plots for resistance to P. salmonis measured as DD in CS, RT, and AS. Y-axis represents the percentage of the GEV by each marker. [file Image_1.tif]

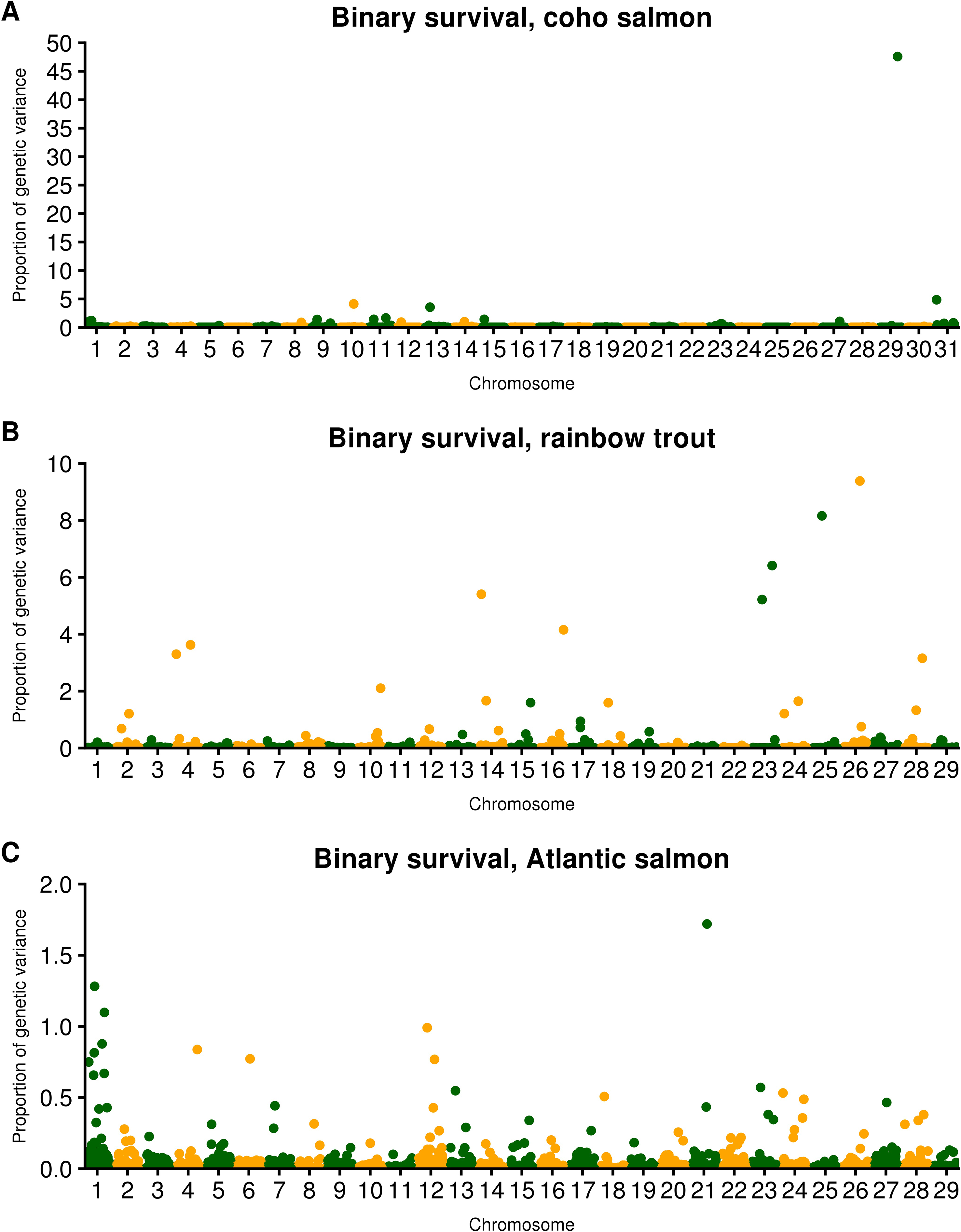

Supplement: Figure S2 — Manhattan plots for resistance to P. salmonis measured as BS in CS, RT, and AS. Y-axis represents the percentage of the GEV by each marker. [file Image_2.tif]

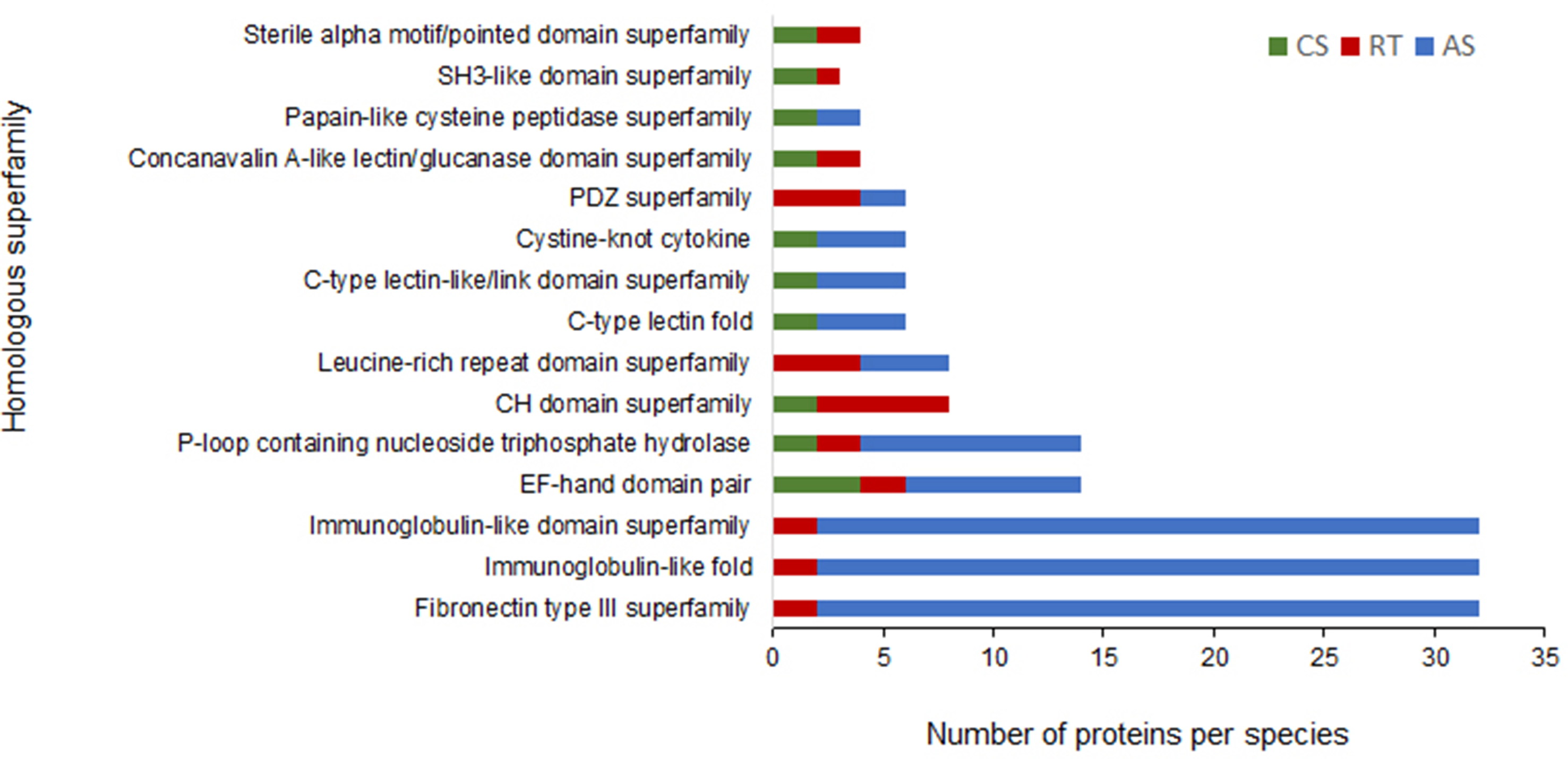

Supplement: Figure S3 — Homologous superfamilies (InterPro) associated with 100 random selected proteins from CS, RT, and AS genomes. [file Image_3.tif]
